# Gene Expression Analysis of Adapted Insect Cells during Influenza VLP Production Using RNA-Sequencing

**DOI:** 10.3390/v14102238

**Published:** 2022-10-12

**Authors:** Marco Silvano, Ricardo Correia, Nikolaus Virgolini, Colin Clarke, Paula M. Alves, Inês A. Isidro, António Roldão

**Affiliations:** 1 IBET, Instituto de Biologia Experimental e Tecnológica, Apartado 12, 2780-901 Oeiras, Portugal; 2 ITQB NOVA, Instituto de Tecnologia Química e Biológica António Xavier, Universidade Nova de Lisboa, Av. da República, 2780-157 Oeiras, Portugal; 3 NIBRT, National Institute for Bioprocessing Research and Training, Fosters Avenue, Blackrock, Co., A94 X099 Dublin, Ireland; 4 School of Chemical and Bioprocess Engineering, University College Dublin, D04 V1W8 Dublin, Ireland

**Keywords:** insect cells, baculovirus expression system, influenza VLP, RNA sequencing, pathway analysis

## Abstract

Adaptive laboratory evolution has been used to improve production of influenza hemagglutinin (HA)-displaying virus-like particles (VLPs) in insect cells. However, little is known about the underlying biological mechanisms promoting higher HA-VLP expression in such adapted cell lines. In this article, we present a study of gene expression patterns associated with high-producer insect High Five cells adapted to neutral pH, in comparison to non-adapted cells, during expression of influenza HA-VLPs. RNA-seq shows a decrease in the amount of reads mapping to host cell genomes along infection, and an increase in those mapping to baculovirus and transgenes. A total of 1742 host cell genes were found differentially expressed between adapted and non-adapted cells throughout infection, 474 of those being either up- or down-regulated at both time points evaluated (12 and 24 h post-infection). Interestingly, while host cell genes were found up- and down-regulated in an approximately 1:1 ratio, all differentially expressed baculovirus genes were found to be down-regulated in infected adapted cells. Pathway analysis of differentially expressed genes revealed enrichment of ribosome biosynthesis and carbohydrate, amino acid, and lipid metabolism. In addition, oxidative phosphorylation and protein folding, sorting and degradation pathways were also found to be overrepresented. These findings contribute to our knowledge of biological mechanisms of insect cells during baculovirus-mediated transient expression and will assist the identification of potential engineering targets to increase recombinant protein production in the future.

## 1. Introduction

The insect cell baculovirus expression vector system (IC-BEVS) relies on infection of insect cells with a recombinant baculovirus genetically modified to include a nucleic acid sequence encoding a gene of interest, commonly under the transcriptional control of very late, strong baculovirus promoters such as *polh* and *p10* [1]. The IC-BEVS system permits the expression recombinant protein at high titers and, importantly, glycosylation patterns similar to those of higher eukaryotes [2]. This expression system is widely used in the production of enzymes, membrane proteins, viral capsids, and envelope proteins for use as vaccines or for analytical purposes [3]. The success of the human papilloma virus vaccine (Cervarix^®^) and the hemagglutinin-based influenza vaccine (Flublok^®^) illustrates the utility of the system for biopharmaceutical manufacturing.

In recent years, academic and industrial research groups have sought to improve knowledge of the molecular characteristics underpinning the efficient production of biopharmaceuticals using IC-BEVS [4]. Understanding the host cell’s metabolic regulation during expression of a foreign gene also facilitates the design and upscaling of a production process [5,6]. Next-generation sequencing technologies have accelerated the development of better expression systems to produce recombinant protein [7]. Transcriptomics was applied to analyze the transcriptional changes of both *Autographa californica* multiple nucleopolyhedrovirus (*Ac*MNPV) [8] and alphanodavirus-free High Five cells (Tnms42) [9] during protein expression using IC-BEVS. It has been shown that the number of viral transcripts increased significantly after the first 6 h of infection, concomitantly with a decrease in expression of host cell transcripts due to global shut-off of the host protein synthesis [10]. More recently, comparative transcriptome analysis was conducted to study the differences in Tnms42 cell response upon expression of intracellular or secreted protein products using IC-BEVS, identifying key proteins as promising targets for achieving higher yields of protein secretion [11].

While genetic engineering of insect cells and/or baculovirus has been used to improve production yields [12], strategies such as shifts in culture parameters to non-physiological values have been shown to impact the growth performance and recombinant protein production [13]. The use of atypical culture conditions in insect cells has also been recently addressed through adaptive laboratory evolution approaches, i.e., by adapting cells to grow at such non-standard culture conditions, allowing higher recombinant protein yields both in stable cell lines [14] and IC-BEVS [15]. In the latter, adaptation of insect High Five cells to grow at neutral pH allowed a threefold improvement in cell-specific production rate of influenza virus-like particles (VLPs). However, relatively little is known about the mechanisms underlying the higher recombinant protein productivity achieved with this adapted cell line.

In this study, the transcriptome of High Five cells adapted to neutral pH (established in [15]), producing influenza VLPs using IC-BEVS, were assessed by RNA-seq and compared to those of parental, non-adapted insect High Five cells in order to gain an understanding of the mechanisms behind the higher productivity of the adapted cell line.

## 2. Materials and Methods

### 2.1. Cell Lines and Culture Media

Insect High Five cells (Invitrogen), hereon referred to as non-adapted cells, and High Five cells adapted to neutral pH [15], hereon referred to as adapted cells, were routinely sub-cultured to 0.3–0.5 × 10^6^ cell.mL^−1^ every 2–3 days when cell concentration reached 2–3 × 10^6^ cell.mL^−1^. Non-adapted cells were cultured in Insect-XPRESS^TM^ medium (Sartorius Stedim Biotech, Göttingen, Germany); adapted cells were cultured in cell culture medium composed of a 1:1 mixture of Insect-XPRESS^TM^ and chemically defined solution as previously reported [14]. Both cell lines were cultured in 125–500 mL shake flasks (10% working volume) and maintained at 27 °C in a Inova 44R shaking incubator (Eppendorf, Hamburg, Germany) set to 100 RPM and with an orbital motion diameter of 2.54 cm.

### 2.2. Baculovirus Amplification and Storage

Recombinant baculoviruses enclosing influenza capsid M1 from A/California/06/2009 H1N1 strain and hemagglutinin (HA) from A/Brisbane/59/2007 strain genes were kindly provided by Redbiotec AG (Schlieren, Switzerland). Amplification of baculovirus stocks was performed as described elsewhere [16].

### 2.3. Production of Influenza HA-VLPs

Influenza HA-VLPs were produced in 500 mL shake flasks (10% working volume) by infecting non-adapted cells and adapted cells at a cell concentration at infection (CCI) of 2 × 10^6^ cell.mL^−1^, with baculovirus using a multiplicity of infection (MOI) of 1 pfu/cell (*n* = 3, number of replicates). Samples were taken daily for the determination of cell concentration and viability, metabolite concentration, and detection/relative quantification of M1 and HA proteins; for RNA-seq, samples were taken before infection, and at 12 and 24 h post-infection (hpi) (further details in Supplementary Figure S1).

### 2.4. Analytics

#### 2.4.1. Cell Concentration and Viability

Cell counting was performed in a Fuchs–Rosenthal hemocytometer chamber (Brand, Wertheim, Germany) and viability was assessed using the trypan-blue exclusion method [17].

#### 2.4.2. Metabolites Concentration

For metabolite quantification, cell culture samples were centrifuged (300× *g*, 4 °C, 5 min) and supernatant collected and stored at −20 °C. Metabolite quantification was performed using Cedex Bio Analyzer 7100 (Roche Diagnostics, Mannheim, Germany).

#### 2.4.3. Baculovirus Titration

Baculovirus titers were determined using the MTT assay as described elsewhere [18,19].

#### 2.4.4. Western Blot

For M1 and HA detection/relative quantification, cell culture samples were centrifuged (300× *g*, 4 °C, 5 min) and supernatant was collected and stored at 4 °C. Western blot analysis was performed as reported elsewhere [14]. Briefly, for HA identification, a mouse monoclonal antibody (IRR, Manassas, VA, USA, FR-494—mouse monoclonal antibody to recombinant H1 HA from influenza A/Brisbane/59/2007 (H1N1)) was used at a dilution of 1:2000, and M1 protein was identified using a goat polyclonal antibody (Abcam, Cambridge, UK, Cat# ab20910) at a dilution of 1:2000. Secondary anti-mouse or anti-goat IgG antibodies conjugated with alkaline phosphatase were used at a dilution of 1:2000 for identification of HA and M1, respectively. Densitometry analysis of Western blot membranes (to assess relative productivity) was performed using the FIJI software [20].

### 2.5. RNA Sequencing and Data Analysis

#### 2.5.1. RNA Isolation and Library Preparation

For RNA sequencing analysis, cell culture samples were centrifuged (300× *g*, 4 °C, 5 min) and pellets collected for RNA extraction using 1 mL of Trizol (Invitrogen) and the Direct-zol RNA mini prep kit (Zymo Research) according to manufacturer’s instructions. RNA purity and quality were assessed by spectrophotometry (mySPEC equipment, VWR) and fragment analysis (Agilent).

Library preparation and sequencing were performed elsewhere (Genewiz, Leipzig, Germany). In short, Poly (A) selection on total RNA (NEBNext^®^ Poly (A) mRNA Magnetic Isolation Module) was performed prior to strand-specific library preparation (NEBNext^®^ Ultra™ II Directional RNA Library Prep Kit for Illumina^®^). Sequencing libraries were quality-checked using Qubit (Invitrogen) and a fragment analyzer (Agilent), and loaded on the Illumina NovaSeq 6000 system configured to yield a minimum of 25 million 2 × 150 bp Paired-End (PE) reads per sample.

#### 2.5.2. Data Processing, Alignment and Counting

Trimmomatic v0.36 was used to remove adapters and to perform quality trimming [21] of the raw RNA-seq reads. The reads were subsequently aligned to a hybrid reference, comprising the insect *Trichoplusia ni* cell genome (Tnl; RefSeq assembly accession: GCF_003590095.1) [22], the baculovirus, i.e., *Ac*MNPV (RefSeq assembly accession: GCF_000838485.1, ViralProj14023) [23], and the transgene (M1 and HA) sequences using STAR v2.7.3a [24]. Those reads mapping to annotated genes were counted using HTSeq [25].

#### 2.5.3. Differential Expression Analysis

The edgeR Bioconductor package [26] was used to determine the number of differentially expressed genes between infected adapted cells and non-adapted cells. Count data were normalized to account for variation in the number of sequenced reads in each sample using the TMM method [27]. To assess differential gene expression, the Fisher’s exact test was used to identify statistically significant differences in gene expression between selected groups [28]. Genes with expression changes of at least 1.5-fold and with a false discovery rate (FDR)-adjusted *p*-value < 0.05 were considered to be differentially expressed.

#### 2.5.4. Functional Annotation and Pathway Enrichment Analysis

For gene annotation, the amino acid sequence of protein-coding genes with at least one read aligned was used as a query. Blastp search was applied in the NCBI nr protein database using Blast2GO OmicsBox software [29]. No taxonomy filter was applied, and the E-value cutoff was set to 1.0 × 10^−3^.

Blast2GO was used to perform pathway enrichment analysis with Fisher’s exact test and the Gene Set Enrichment Analysis (GSEA) method [30].These tests were used to identify statistically significant over-represented biological processes for each differentially expressed gene list. FDR was applied as multiple test correction method with a cut-off of 0.05 [31]. 

## 3. Results

### 3.1. Production of Influenza HA-VLPs Using Adapted Insect High Five Cells

**Figure 1:**
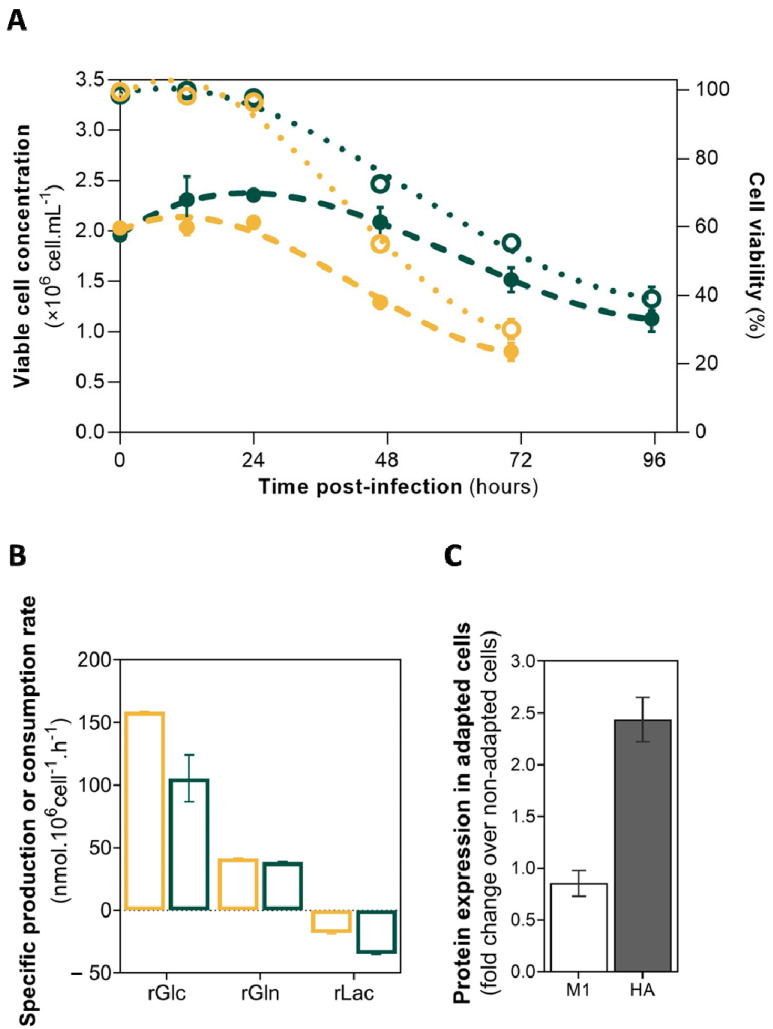
HA-VLP production in non-adapted and adapted High Five insect cells using the baculovirus expression vector system. (**A**) Viable cell concentration (full circles) and cell viability (empty circles) after infection. (**B**) Specific glucose (rGlc) and glutamine (rGln) consumption and lactate (rLac) production (hence shown as negative) rates, estimated by linearization of metabolite concentration and integral of total cells, during infection. (**C**) Fold-change (adapted/non-adapted) in expression of HA and M1 proteins assessed by densitometry analysis of Western blot images (i.e., relative band intensity). Color code: yellow represents non-adapted cells, green represents adapted cells. Data are expressed as mean ± standard deviation of three culture replicates (*n* = 3).

Adapted and non-adapted cells were infected at the optimum conditions (CCI of 2 × 10^6^ cell.mL^−1^ and MOI of 1 pfu.cell^−1^) previously identified in our laboratory [14], and infection kinetics and HA expression were assessed throughout. Adapted cells maintained higher cell concentration and viability upon infection, with the onset of cell viability drop having a 24 h delay in comparison with non-adapted cells (Figure 1A). The specific consumption or production rates for glucose (Glc), glutamine (Gln), and lactate (Lac) were similar in both cell lines, with the highest variation observed for glucose (Figure 1B). Importantly, M1 and HA proteins were identified by Western blot (Supplementary Figure S2), with relative band intensities suggesting that expression of HA was approximately 2.5-fold higher in adapted cells while M1 expression remained similar in both cell lines (Figure 1C), in agreement with previously reported data [15].

### 3.2. Gene Expression Profiling of Infected Adapted vs. Non-Adapted Cells

To reveal the differences at the transcriptional level between adapted and non-adapted cells upon infection, quality-filtered reads were mapped to the reference genome and sequences. A total of 10,850 and 10,987 reads (at 12 hpi), and 10,082 and 10,236 reads (at 24 hpi) were mapped in samples from adapted and non-adapted cells, respectively; from these, 10,389 (at 12 hpi) and 9620 (at 24 hpi) reads are coincident in both cell lines.

**Figure 2:**
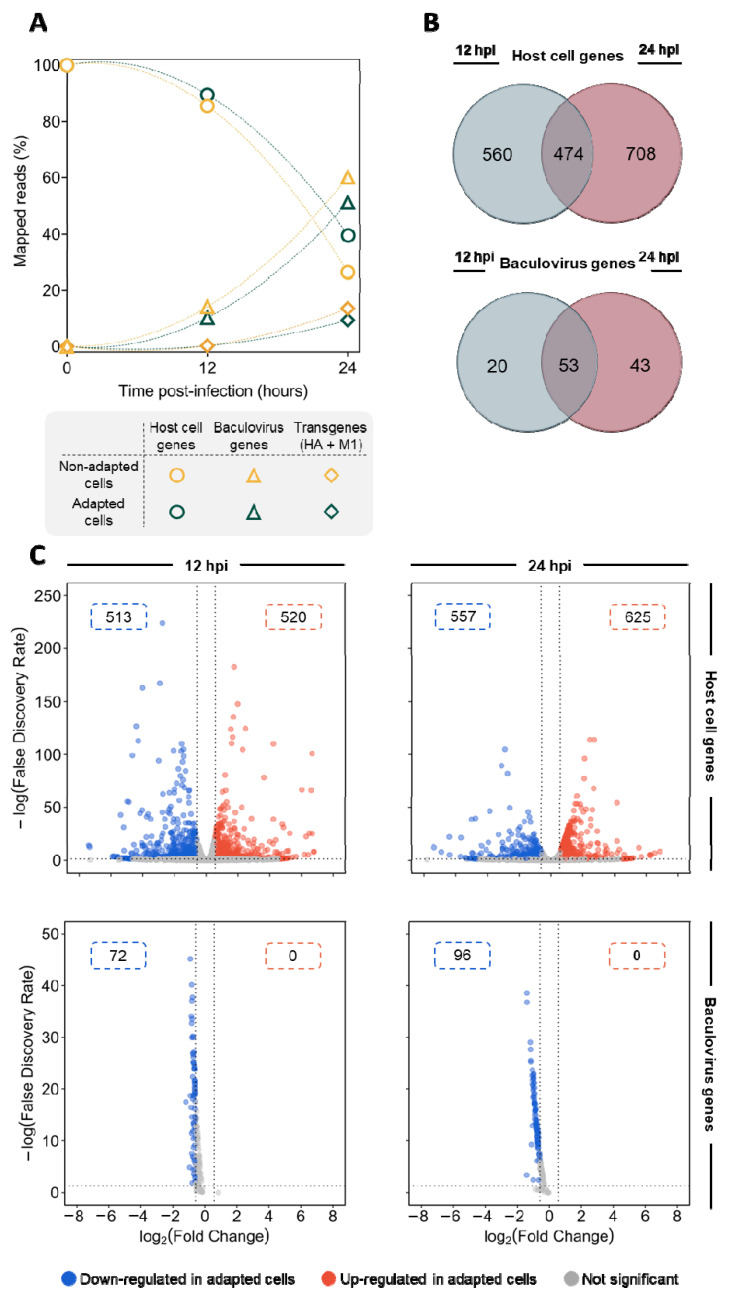
Differential gene expression analysis. (**A**) Percentage of reads mapping to host cell (circles), baculovirus (triangles) and transgenes (diamonds) throughout infection. (**B**) Venn diagram showing the number of host-cell and baculovirus genes being differentially expressed (|log2 (Fold-Change| > 0.58 and FDR < 0.05) between adapted and non-adapted cells, at 12 hpi, 24 hpi, and both timepoints simultaneously. (**C**) Volcano plot showing the distribution of differentially expressed genes with higher (in red) or lower (in blue) expression in adapted cells, compared to non-adapted cells. Number in boxes: number of differentially expressed genes.

A reduction in the proportion of reads mapping to the host cell genome was observed between 12 and 24 hpi, with a concomitant increase in reads mapping to baculovirus and transgenes; at 24 hpi, 51–60% of the total reads were mapped to baculovirus genome and 9–14% of the total reads were mapped to transgenes (HA + M1) (Figure 2A). Differential gene expression analysis revealed that a total of 1742 host cell genes were differentially expressed between adapted and non-adapted cells throughout infection, 474 of those being differentially expressed at both time points evaluated (Figure 2B). The top 20 most differentially expressed host cell genes at both 12 and 24 hpi are detailed in Table 1 and Table 2. The most up-regulated genes encode for transmembrane transports (e.g., *organic cationic transports protein-like*, 93-fold at 24 hpi) and proteins involved in proteolysis (e.g., *xxa-Pro aminopeptidase 1*, 756-fold at 24 hpi), and lipase activity (e.g., *lipase member H-like*, 112-fold at 12 hpi), whereas the most down-regulated genes encode for proteins involved in programmed cell death (e.g., *homeobox protein abdominal-A homolog isoform X1*, 120-fold at 24 hpi), lipid transport (e.g., *apolipophorins*, 50-fold at 24 hpi), innate immune response (e.g., *protein toll-like*, 37-fold at 12 hpi), and oxidoreductase (e.g., *cytochrome P450 9e2-like,* 163-fold at 12 hpi). Protein-coding genes involved in the regulation of transcription and signaling were found both up- and down-regulated. While host cell genes were found up- and down-regulated in an approx. 1:1 ratio regardless of infection time, all baculovirus differentially expressed genes were found down-regulated in infected adapted cells (Figure 2C).

### 3.3. Pathway Enrichment Analysis

**Figure 3:**
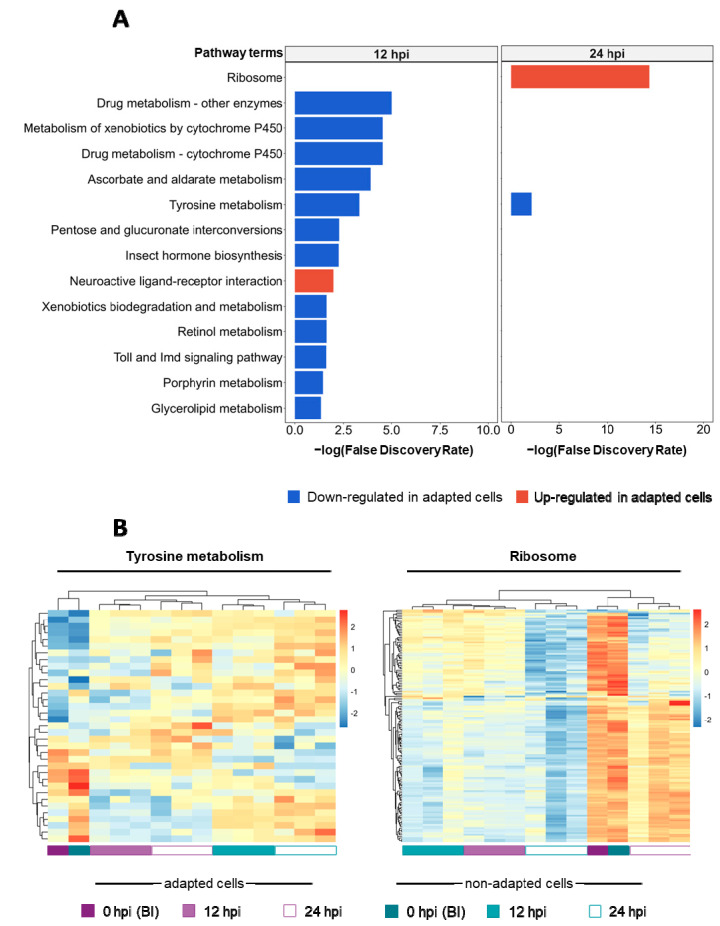
Pathway enrichment analysis. (**A**) Pathway enrichment analysis of differentially expressed genes using Fisher’s exact test. Bar plots indicate enriched terms at 12 and 24 hpi (i.e., longer bars denote pathways more significantly enriched). Color code: red identifies pathways found up-regulated and blue identifies pathways found down-regulated in adapted cells, over non-adapted cells. (**B**) Heat map of tyrosine metabolism pathway and genes encoding for ribosome subunits; the z-score (gradient color code) is defined for all the genes found to be involved in these specific pathways (differentially expressed or not). BI denotes before infection.

To further understand the biological mechanisms behind adapted cells’ higher productivity, pathway enrichment analysis was performed. KEGG analysis using Fisher’s exact test revealed that 14 pathways are enriched, two being up-regulated in adapted cells (i.e., neuroactive ligand-receptor interaction, ribosome) while the remaining 12 were down-regulated (including metabolism of xenobiotic and endogenous compounds, carbohydrates, amino acids, vitamins and lipids) (Figure 3A). Additional pathways were found to be enriched using the GSEA method, including those playing a role in protein processing (including proteasome and ubiquitin mediated proteolysis) and oxidative phosphorylation (Supplementary Figure S3). For the pathways found to be enriched, heatmaps with hierarchical clustering of all genes (differentially expressed and not) were generated. Results suggest that there is no common trend in adapted and non-adapted samples clustering as infection progresses, as reflected in the different dendrograms obtained—see two examples in Figure 3B.

## 4. Discussion

In this work, we assessed the gene expression profile of insect High Five cells adapted to neutral pH during production of influenza HA-VLPs using IC-BEVS, and compared it to that of non-adapted cells.

During infection, the number of reads mapping to the host cell genome decreased, whereas those aligned to baculovirus (*Ac*MNPV) and transgene (M1 and HA) sequences increased. Such a trend is a consequence of the global takeover of the cellular transcription machinery by baculoviruses towards overproduction of viral proteins during infection [4]. The percentages of reads aligned to host cells and virus genome throughout the course of infection is similar to those reported previously in other studies [9,11]. Differential expression analysis revealed that all baculovirus-derived genes were found to be down-regulated in adapted cells. Together with distinct onset cell viability drop, this suggests that adapted and non-adapted cells have different susceptibility to infection.

Adapted and non-adapted cells respond differently to baculovirus infection as reflected by the major differences observed in their gene expression profiles. For instance, genes involved in lipid metabolism processes (i.e., biosynthesis, hydrolysis, and transport) were found to be either up- or down-regulated in adapted cells at both 12 and 24 hpi. Insect cells are known to have a limited lipid metabolism, reflected in their limited capacity in synthesizing, desaturating and elongating fatty acids [32], and lipid deprivation is linked to cell degeneration and impairment in the production of baculovirus [33]. Moreover, lipids such as cholesterol are especially important during the production of enveloped viral particles in mammalian culture systems [34] and IC-BEVS [35], such as the case of the influenza HA-VLPs herein produced. Therefore, distinct lipid metabolism could be associated with different productivity of adapted and non-adapted cells.

Pathway enrichment analysis revealed that genes encoding ribosome subunits were up-regulated in adapted cells at 24 hpi. Since baculovirus is known to promote the shutdown of host cell protein synthesis upon infection for overproduction of viral proteins [10,36], this result suggests that the host cell translation machinery was less impaired in adapted cells, thereby leading to higher protein biosynthesis (including of HA-VLPs). The oxidative phosphorylation pathway was also found to be up-regulated in adapted cells at 24 hpi, allowing lower substrate consumption when compared to non-adapted cells. In contrast, pathways associated with drug, carbohydrate, and amino acid metabolism were down-regulated in adapted cells. Interestingly, the set of genes driving such enrichment is shared between most pathways; these code for proteins such as *UPD-glucuronosyltransferases-like* and *glutathione S-transferase-like* proteins, enzymes associated with cellular protection and resistance to oxidative stress [37]. In addition to these, genes involved in oxidative metabolism were also down-regulated in adapted cells, some of which have been already identified as differentially expressed in insect cells resistant to harmine and fungi (e.g., *cytochromes 450*) [38,39]. Pathways associated with baculovirus infection such as immune response, protein processing in the endoplasmic reticulum, proteosome and ubiquitin mediated proteolysis, which are known to be up-regulated upon infection [4,40], were also found down-regulated in adapted cells. Taken together, these results suggest that adapted cells cope better with the stress induced by baculovirus infection when compared to non-adapted cells.

The major *Ac*MNPV fusion protein, GP64, plays an essential role in mediating virus–receptor binding, internalization, and membrane fusion during virus entry into both mammalian and insect cells [41]. The fusogenicity of GP64 is low-pH-dependent, and fusion of the viral envelope with the endosomal membrane is triggered in the acidic endosomal lumen [42]. Virus fusion was shown to be impaired at relatively high-pH conditions in mammalian cells [43]; thus, baculovirus entry could be less efficient in adapted cells. In this regard, the baculovirus gene *ACNVgp64* was found to be significantly down-regulated in adapted cells at 12 hpi (1.7-fold) and 24 hpi (2.0-fold). While the amount of differentially expressed host cell genes in adapted cells was equally distributed between those being up- or down-regulated, all the differentially expressed baculovirus-derived genes herein identified were shown to be down-regulated in adapted cells, hence showing that virus transcripts are produced earlier (or at higher quantity) in non-adapted cells. Whether this outcome is a consequence of less efficient virus entry in adapted cells still remains unknown, but such a fact could be behind the different cell growth kinetics observed for adapted cells, i.e., a slight increase in cell concentration and delayed onset of cell viability drop. The apparent lower burden caused to adapted cells at initial stages of infection may have been the key to achieving prolonged infection and consequently higher productivity.

## 5. Conclusions

In this study, comparative transcriptome analysis revealed significant differences between adapted and non-adapted insect High Five cells during production of influenza HA-VLPs using IC-BEVS. Differential gene expression analysis showed baculovirus genes being down-regulated in adapted cells, revealing less susceptibility to infection. Several pathways were found enriched and differently regulated, such as those associated with protein synthesis, metabolism of xenobiotic and endogenous compounds, carbohydrates and amino acids. The gene expression signatures herein identified can be exploited for rational genetic engineering of insect cells and/or baculovirus to further improve production yields. Furthermore, single-cell RNA sequencing could help us to further understand the molecular signatures playing a role in systems’ productivity, as well as to conclude on adapted cell population heterogeneity.

## Figures and Tables

**Table 1:** List of TOP 20 up-regulated genes in adapted cells vs. non-adapted cells.

Gene ID	Gene Name	hpi	FC	logCPM	Biological Process (P) or Molecular Function (M)
LOC113500835	*xaa-Pro aminopeptidase 1*	12	273.6	3.1	hydrolase activity (M)
		24	756.1	1.6	
LOC113493104	*potassium channel subfamily K member 18-like*	12	102.1	1.7	transmembrane transport (P)
		24	433.4	0.8	
LOC113505620	*serine protease HP21 precursor*	12	104.1	−0.1	proteolysis (P)
		24	116.2	−0.9	
LOC113496461	*lipase member H-like*	12	111.6	−1.6	lipid metabolic process (P)
		24	79.4	−1.3	
LOC113506781	*organic cation transporter protein-like*	12	91.9	−0.3	transmembrane transport (P)
		24	92.8	−1.1	
LOC113504337	*GATA zinc finger domain-containing protein 4-like*	12	79.8	−0.5	regulation of transcription (P)
		24	74.3	−1.3	
LOC113500515	*alkylglycerol monooxygenase-like*	12	97.8	1.0	lipid metabolic process (P)
		24	46.6	−0.3	
LOC113501396	*transcription factor glial cells missing 2-like*	12	108.6	−1.7	regulation of transcription (P)
		24	29.1	−2.2	
LOC113501938	*monocarboxylate transporter 1-like*	12	86.2	−1.8	transmembrane transport (P)
		24	36.5	−1.9	
LOC113494762	*octopamine receptor Oamb isoform X1*	12	65.3	1.1	regulation of transcription (P)
		24	18.4	0.7	
LOC113495982	uncharacterized protein	12	7.7	−1.0	n.a.
		24	74.8	−1.3	
LOC113501619	*organic cation transporter protein-like*	12	43.4	−2.5	transmembrane transport (P)
		24	24.1	−2.3	
LOC113506861	uncharacterized protein	12	37.4	−2.6	n.a.
		24	24.0	−2.3	
LOC113492056	*glucose dehydrogenase [FAD, quinone]-like*	12	34.6	−2.7	oxidoreductase activity (M)
		24	26.4	−2.2	
LOC113495496	*thyrotropin-releasing hormone receptor-like isoform X1*	12	3.8	−0.2	signaling (P)
		24	51.7	1–6	
LOC113500144	uncharacterized protein	12	39.2	−0.2	n.a.
		24	14.2	−1.6	
LOC113506931	*proton-coupled amino acid transporter-like protein pathetic*	12	36.3	−1.4	transmembrane transport (P)
		24	6.1	−1.2	
LOC113500825	*acid sphingomyelinase-like phosphodiesterase 3a*	12	4.5	−0.9	hydrolase activity (M)
		24	34.2	−2.1	
LOC113498260	*mitochondrial import receptor subunit TOM40 homolog*	12	29.8	0.3	transmembrane transport (P)
		24	7.7	−0.6	
LOC113500192	*glutamate receptor ionotropic, kainate 2-like*	12	18.8	3.0	regulation of transcription (P)
		24	18.4	2.2	

hpi: hours post-infection; FC: Fold-Change (adapted/non-adapted); CPM: Copy Per Million; n.a.: not available.

**Table 2:** List of TOP-20 down-regulated genes in non-adapted vs. adapted cells.

Gene ID	Gene name	hpi	FC	logCPM	Biological Process (P) or Molecular Function (M)
LOC113496224	*cuticle protein 16.5-like*	12	168.3	−1.0	structural molecule activity (M)
		24	84.3	−1.4	
LOC113492802	*juvenile hormone esterase-like*	12	11.5	−0.1	hydrolase activity (M)
		24	165.3	−0.5	
LOC113495404	*cytochrome P450 9e2-like*	12	163.0	−1.1	oxidoreductase activity (M)
		24	13.6	−1.3	
LOC113496454	*homeobox protein abdominal-A homolog isoform X1*	12	41.7	0.7	programmed cell death (P)
		24	120.1	−0.9	
LOC113508341	uncharacterized protein	12	31.2	1.2	n.a.
		24	83.5	0.2	
LOC113498352	*apolipophorins isoform X2*	12	16.2	1.3	lipid transport (P)
		24	49.9	0.2	
LOC113493958	*solute carrier family 15 member 1-like*	12	36.2	−1.3	transmembrane transport (P)
		24	26.3	−2.3	
LOC113496890	*Acanthoscurrin-1-like*	12	25.3	−1.7	n.a.
		24	34.5	−2.1	
LOC113501789	uncharacterized protein	12	19.4	2.7	anatomical structure development (P)
		24	31.2	1.3	
LOC113499613	*gloverin-like*	12	21.3	2.9	n.a.
		24	26.4	1.5	
LOC113503497	*chondroitin proteoglycan 2-like*	12	14.2	−0.7	chitin binding (M)
		24	31.3	−1.0	
LOC113496839	*protein toll-like*	12	36.6	0.3	immune system process (P)
		24	8.8	−1.3	
LOC113492804	*esterase FE4-like*	12	29.1	1.3	hydrolase activity (M)
		24	15.1	0.5	
LOC113494776	*myb-like protein D*	12	7.0	−2.3	n.a.
		24	34.2	−2.1	
LOC113494175	*alpha-tocopherol transfer protein-like*	12	12.7	−1.0	n.a.
		24	25.3	−2.3	
LOC113503288	*acetylcholine receptor subunit alpha-like 1*	12	23.4	−0.3	signaling (P)
		24	14.4	−0.8	
LOC113507593	*probable E3 ubiquitin-protein ligase bre1 isoform X1*	12	6.1	−1.0	n.a.
		24	30.2	−1.0	
LOC113503500	*odorant receptor 67c-like*	12	4.7	−1.8	signaling (P)
		24	31.3	−2.2	
LOC113505115	*irregular chiasm C-roughest protein-like isoform X1*	12	16.5	−0.3	programmed cell death (P)
		24	15.3	−1.7	
LOC113506962	*neural retina-specific leucine zipper protein-like*	12	25.1	2.3	regulation of transcription (P)
		24	5.5	1.1	

hpi: hours post-infection; FC: Fold-Change (non-adapted/adapted); CPM: Copy Per Million; n.a.: not available.

## Data Availability

The sensitive nature of some of the reagents used in this study (e.g., cell lines, plasmids, baculoviruses, and antibodies) means that they are only readily available internally to the author’s institutions staff for the R&D purposes. For external researchers, approval of reagents request may be obtained via e-mail addressed to the corresponding author.

## Supplementary Materials

The following supporting information can be downloaded at: https://www.mdpi.com/article/10.3390/v14102238/s1, Figure S1: Experimental design; Figure S2: Identification of HA and M1 proteins by western blot; Figure S3: Pathway enrichment analysis using the GSEA method.
