# Supplementary material for: Gene Expression Analysis of Adapted Insect Cells during Influenza VLP Production Using RNA-Sequencing
Source: Viruses. 2022 Oct 12;14(10):2238. doi: 10.3390/v14102238 (PMC9609815; doi:10.3390/v14102238)
Supplement: Supplementary file 1 [file viruses-14-02238-s001.zip › viruses-1939226-supplementary.pdf]

Figure S1

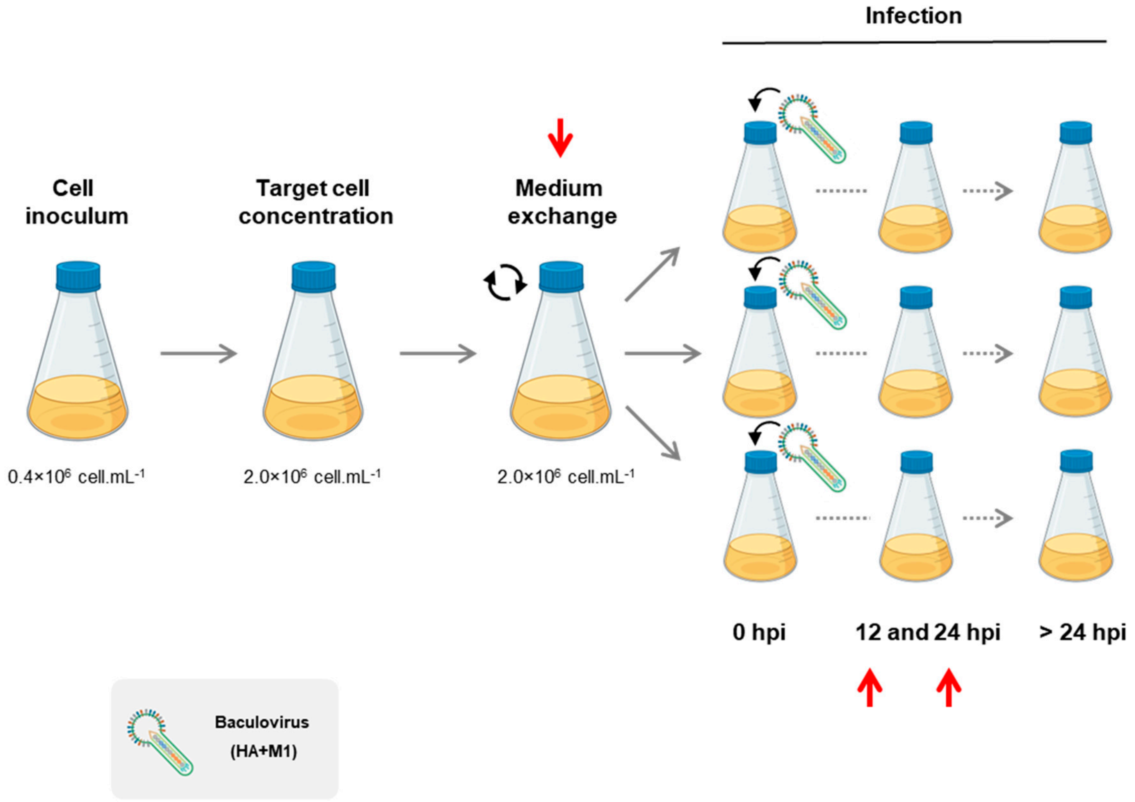

Figure S1. Experimental design. Cells were inoculated at 0.4×10^6 cell.mL^{-1} and grown until the target cell concentration of 2.0×10^6 cell.mL^{-1}. Medium exchange was performed before the infection (using centrifugation), and cells divided in three shake flasks. The red arrows highlight time-points at which samples were collected for total RNA extraction. hpi denoted hours post-infection.

Figure S2

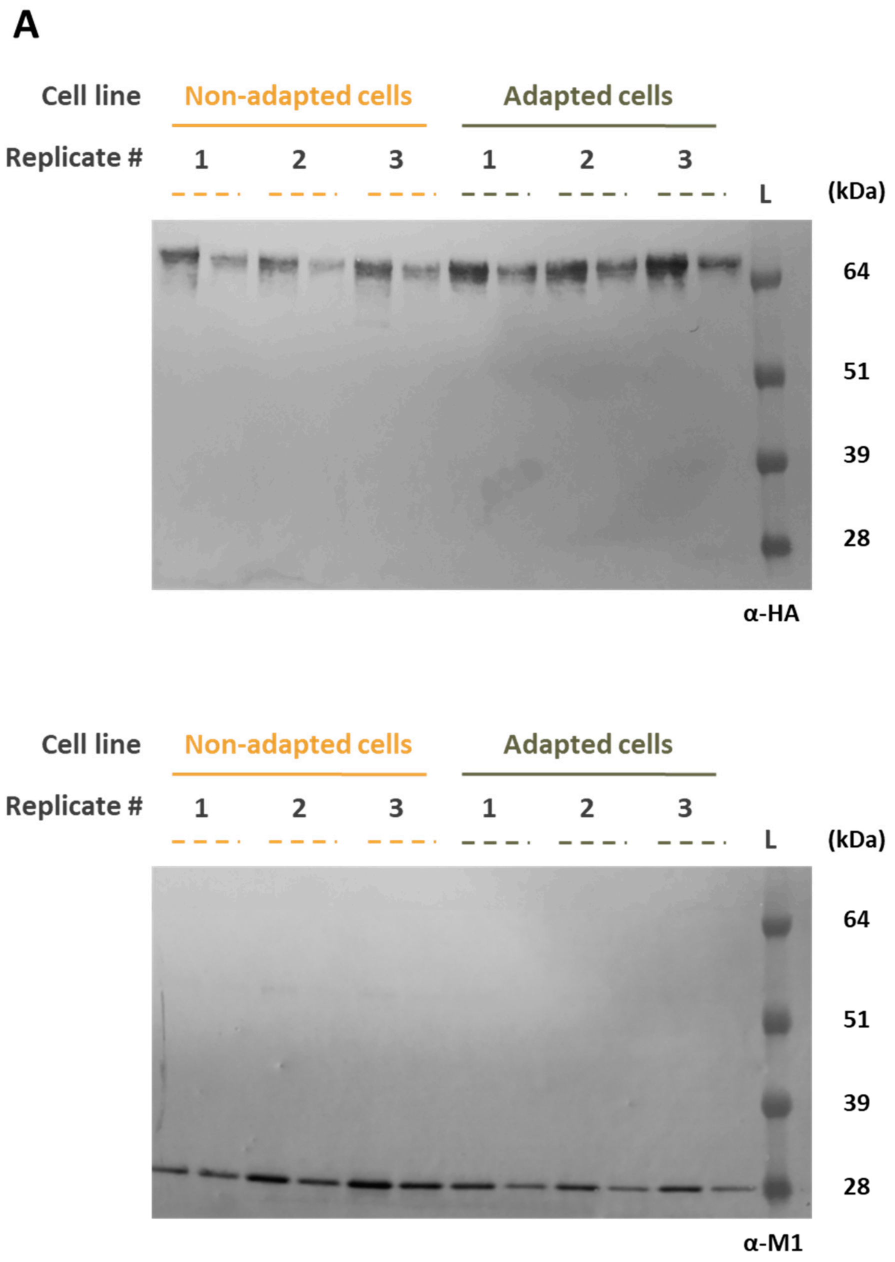

Figure S2. Identification of HA and M1 proteins by western blot. Two different dilutions of in-process samples collected from each cell culture (in triplicate) at the time of harvest are represented. Ac denotes adapted cells, NAc denotes non-adapted cells.

Figure S3

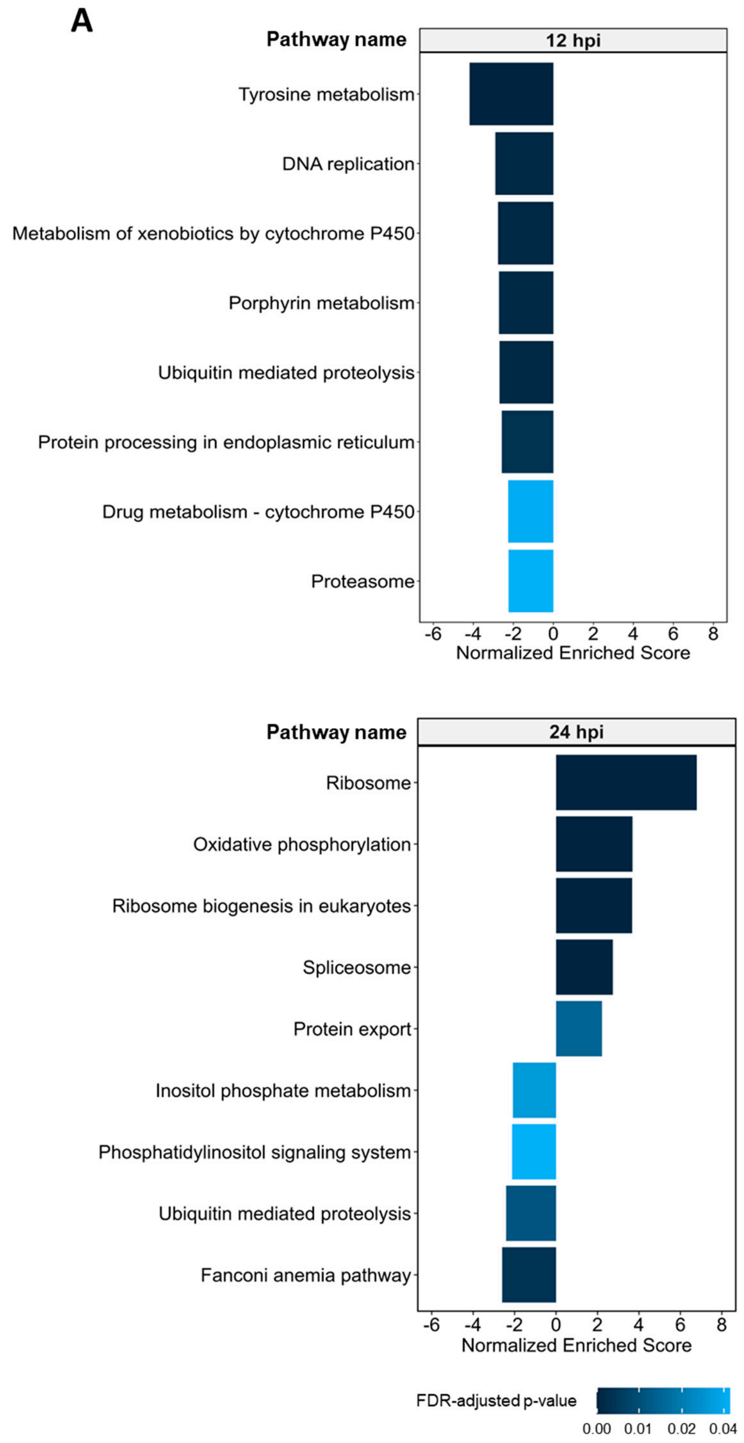

Figure S3. Pathway enrichment analysis using the GSEA method. Barplots show enriched terms at 12 hpi and 24 hours post-infection (hpi). Color gradient of bars indicate the False Discovery Rate (FDR)-adjusted p-value.
